# Neurocritical care update

**DOI:** 10.1186/s40560-016-0141-8

**Published:** 2016-05-28

**Authors:** Yasuhiro Kuroda

**Affiliations:** Department of Emergency, Disaster, and Critical Care Medicine, Faculty of Medicine, Kagawa University, 1750-1, Ikenobe, Miki, Kita, Kagawa Japan 761-0793

**Keywords:** Neurocritical care, Electroencephalogram monitoring, Shivering, Nonconvulsive status epilepticus, Target temperature management, Sepsis-associated brain dysfunction, Review

## Abstract

This update comprises six important topics under neurocritical care that require reevaluation. For post-cardiac arrest brain injury, the evaluation of the injury and its corresponding therapy, including temperature modulation, is required. Analgosedation for target temperature management is an essential strategy to prevent shivering and minimizes endogenous stress induced by catecholamine surges. For severe traumatic brain injury, the diverse effects of therapeutic hypothermia depend on the complicated pathophysiology of the condition. Continuous electroencephalogram monitoring is an essential tool for detecting nonconvulsive status epilepticus in the intensive care unit (ICU). Neurocritical care, including advanced hemodynamic monitoring, is a fundamental approach for delayed cerebral ischemia following subarachnoid hemorrhage. We must be mindful of the high percentage of ICU patients who may develop sepsis-associated brain dysfunction.

## Introduction

Neurocritical care is the intensive care provided to patients with severe neurological and neurosurgical conditions. It provides the interface between the brain and other organ systems. Neurocritical care provides comprehensive medical and specialized neurological support for patients with life-threatening neurological diseases by integrating and balancing the management of both the brain and the body [1]. The aim of this review is to provide an update on neurocritical care in adults.

## Review

### Post-cardiac arrest brain injury and targeted temperature management

#### Overview

Post-cardiac arrest brain injury is a syndrome of acute global brain injury resulting from a critical reduction in blood flow or oxygen and nutrient supply. Its most common clinical features include disorders pertaining to consciousness (coma and vegetative status), seizures, and myoclonus. Targeted temperature management (TTM; 32–36 °C) and diagnosing/treating seizures are essential elements of post-resuscitation care for global ischemic brain injury. Furthermore, careful evaluation and management of other organ injuries (myocardial dysfunction, aspiration pneumonia, bowel ischemia, acute kidney injury, and disordered glucose regulation) is required. Hematologic and coagulation disorders are also recognized to be associated with post-cardiac arrest syndrome (PCAS) [2]; however, they are not well described and fully understood.

Regarding the temperature control, the term “TTM” is recommended to emphasize the importance of defining a complete temperature profile [3]. The specific temperature ranges of TTM (32–36 °C) include therapeutic hypothermia (TH; 32–34 °C) and fever control (normothermia, approximately 36 °C).

#### Optimal targeted temperature

TH (32–34 °C) [4], according to the positive neuroprotective results of randomized controlled trials (RCTs) [5, 6], is recommended for comatose (i.e., lack of meaningful response to verbal commands) adult patients who achieve return of spontaneous circulation (ROSC) after out-of-hospital cardiac arrest. However, a recently published RCT concluded that in comatose survivors or in those who sustained out-of-hospital cardiac arrest, normothermia (36 °C) management provided the same benefits as those provided by TH (33 °C) [7]. A reason for this discrepancy is the differences in the study design [8] of TTM trial [7]: larger deviation in body temperature, neurological evaluation performed 72 h (too early) after the intervention, and relatively low incidence of favorable neurological outcome [9]. Another reason for this discrepancy may be that the severity of organ injury, including the brain, varies among studies and patients because of the absence of an established modality enabling proper evaluation.

Optimal target temperature during TTM corresponding to the post-cardiac arrest brain injury remains to be evaluated, although 32–36 °C has been generally adopted [5–7]. A small pilot RCT comparing 32 and 34 °C management found that 32 °C management has benefits pertaining to neurologically intact survival [10]. A recent study [11] showed that PCAS patients with a resuscitation interval of <30 min may be candidates for TH using a target temperature of <34 °C. Regardless of the target temperature, temperature control remains a key aspect in the management of post-cardiac arrest patients [12]. If a temperature of 36 °C is selected, shivering is likely to be more pronounced because the patients’ thermoregulatory defenses, which are partly suppressed at 32–33 °C, will be much more active at 36 °C [13]. Specific target temperature interventions tailored for individual patients await further research.

#### Evaluation of brain damage

To date, the degree of damage in the brain and other organs of TH candidates has been estimated using combinations of several indirect factors, such as bystander cardiopulmonary resuscitation, witness, initial rhythm, and downtime [5–7]. Moreover, brain damage after ROSC varies among patients despite their comatose status [14].

Although there have been three studies [7, 14, 15] reporting on the admission Glasgow Coma Score (GCS) motor score as a measure of the efficacy of TH in comatose cardiac arrest survivors, the association between GCS motor score and neurologic outcome remains unknown. Two recent studies examined the GCS motor scores immediately after ROSC (day 0), and the outcome suggested that the score is an independent predictor of good neurologic outcome [16] and that no significant differences of neurologic outcome at 30 days after hospital admission was observed between mild TH and control in the subgroup of GCS motor score 5 or 6 [17, 18]. These data show that initial GCS motor score examination immediately after ROSC can at least provide baseline objective prognostic data for decisions by healthcare professionals.

The full outline of unresponsiveness (FOUR) score includes additional information not assessed by GCS, including brain stem reflex, visual tracking, breathing patterns, and respiratory drive (Table 1) [19]. An early, novel illness severity score using FOUR and serial organ function assessment scores at hospital or intensive care unit (ICU) arrival predicts outcome after cardiac arrest [20, 21].

**Table 1:** FOUR score [19] with permission

Eye response	
4	Eyelids open or opened, tracking, or blinking to command
3	Eyelids open but not tracking
2	Eyelids closed but open to loud voice
1	Eyelids closed but open to pain
0	Eyelids remain closed with pain
Motor response	
4	Thumbs-up, fist, or peace sign
3	Localizing to pain
2	Flexion response to pain
1	Extension response to pain
0	No response to pain or generalized myoclonus status
Brainstem reflexes	
4	Pupil and corneal reflexes present
3	One pupil wide and fixed
2	Pupil or corneal reflexes absent
1	Pupil and corneal reflexes absent
0	Absent pupil, corneal, and cough reflex
Respiration	
4	Not intubated, regular breathing pattern
3	Not intubated, Cheyne–Stokes breathing pattern
2	Not intubated, irregular breathing
1	Breathes above ventilator rate
0	Breathes at ventilator rate or apnea

*FOUR* full outline of unresponsiveness

Shivering may reflect the degree of brain damage. Data from a recent study shows that patients who experienced shivering (60 %) because of the induction of TH for PCAS had a significantly better rate of favorable (cerebral performance categories 1–2) neurologic outcome at discharge from the hospital compared to patients who did not experience shivering (36 %) [22]. Conversely, no shivering after the induction of hypothermia or spontaneous hypothermia prior to the induction of hypothermia has been associated with a poor outcome [23]. Shivering may be a sign of a less-impaired thermoregulatory pathway. Neurological signs such as GCS, brain stem reflex, respiratory status, and degree of shivering are potential variables that can be incorporated into a predictive model for a more precise evaluation of brain injury in cardiac arrest survivors undergoing TTM.

### Analgosedation for the targeted temperature management

#### Prevention of shivering during therapeutic hypothermia

Shivering is a centrally mediated thermoregulatory response that usually initiates at approximately 35.5 °C and is overcome below 34 °C. The management and prevention of shivering is an important consideration during TTM therapy. With preventing shivering and further facilitating the induction of TTM, sedation and analgesia play an important role in optimizing ventilator synchrony and minimizing endogenous stress induced by catecholamine surges. Inadequate sedation that allows shivering is the most common cause of failure in achieving or maintaining the target temperature.

**Fig. 1:**
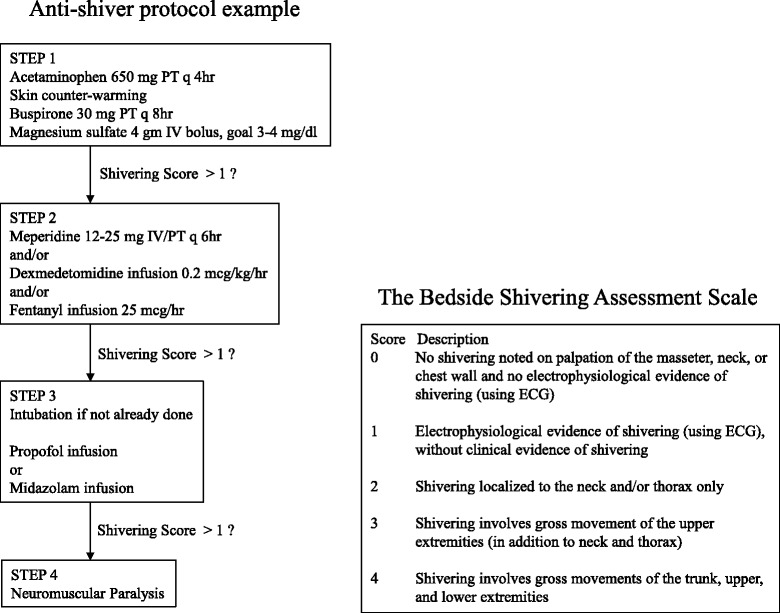
Anti-shivering protocol example and the bedside shivering assessment scale. *IV* intravenous, *PT* per feeding tube, *ECG* electrocardiogram. Modified from Brophy [25] and Badjatia [24] with permission

Shivering is suppressed using a stepwise protocol during TTM, and a clinical scale is used to quantify and assess it. A suggested protocol based on the measurement of the shivering index has been reported (Fig. 1) [24, 25]. However, there is no clear evidence to identify the preferred sedation and analgesia agents in TTM. Skin counter-warming (warming of the non-cooled areas of the skin) should be considered even when surface cooling methods are used [13]. It was achieved by covering the anterior surface (the hands, feet, and face) of the patients with an air circulating blankets warmed to the maximal temperature setting (43 °C) to reduce shivering [26]. Shivering can be managed without the use of paralytic agents in most patients in the setting in ICU [13].

In comparison with benzodiazepines, propofol and dexmedetomidine have desirable properties when continuous infusions of sedative agents are required [27]. Doses of both agents should be titrated to the desired effect, and the rate of infusion should be accordingly adjusted. In addition, the need for continuous infusions during the maintenance phase, when shivering is less likely to occur, should be evaluated [27]. If fentanyl is used in the maintenance phase of TTM, intensivists should be aware that the rate of fentanyl elimination in humans does not trend toward normal for at least 8 h during rewarming [28].

Shivering following rewarming because of post-cardiac arrest TTM is known to increase body temperature (rebound pyrexia); marked pyrexia (median >38.7 °C) was associated with a significantly lower proportion of favorable outcome survivors (58 vs. 80 %) [29]. The addition of a period of fever control (normothermia) subsequent to TTM rewarming should be evaluated.

#### Neuromuscular blockade

The benefits and risks of neuromuscular blockade (NMB) during TTM are controversial. Uncontrolled shivering during TTM has been associated with a longer time to achieve the target temperature; thus, uncontrolled shivering may adversely affect outcomes in cardiac arrest survivors. NMB administration in TH not only decreases refractory shivering but also facilitates both the rapid achievement and maintenance of the target temperature. A recent report suggests that continuous intravenous NMB therapy has a beneficial effect on the survival of patients undergoing post-cardiac arrest TH [30]. However, the same study also reported that NMB therapy is associated with a non-significant increase in the frequency of early-onset pneumonia [30]. Although continuous intravenous NMB is associated with decreased mortality according to a study [31], the association between NMB, TH, and outcomes must be further analyzed in terms of shivering score (Fig. 1), NMB usage, complications, and degree of brain damage [32].

However, NMB obscures convulsive activity, which may be an important component in the neurological evaluation. Continuous electroencephalography (EEG) should be considered in comatose post-cardiac arrest patients, particularly if NMB is used [4]. In TTM, routine and/or continuous NMB use is not supported [13] and the duration of NMB should be kept to a minimum or avoided altogether [4]. On the basis of pharmacokinetics of NMB, it would be reasonable to use intermittent dosing with the lowest effective dose during TTM to avoid overdosing upon rewarming.

### Traumatic brain injury

#### Therapeutic hypothermia

Traumatic brain injury (TBI) may cause disability and death because of a combination of primary (shearing damage to the neurons or glial cells at the time of impact) and secondary (ischemia/hypoxia and reperfusion injury) brain injuries [33]. For TBI, the specific effects of TH include limiting the secondary brain injury by not only reducing intracranial pressure (ICP) and cerebral metabolic demands but also decreasing the disruption of the blood–brain barrier, inhibiting the inflammatory cytokines, and reducing free radicals related to reperfusion injury [34–37].

Clinical trials have been conducted to investigate the effects of mild TH (32–34 °C) on TBI, but they could not demonstrate more favorable outcomes than those demonstrated by the trials conducted to investigate the effects of normothermia (37 °C) [38–40]. In Japan, a multicenter RCT (brain hypothermia therapy for acute head injury (BHYPO)) was conducted in patients with severe TBI who received either mild TH (32.0–34.0 °C) or underwent fever control (35.5–37.0 °C). The protocol was well designed to improve former considerations such as prolonged mild TH (more than 72 h), tight hemodynamic monitoring, and slow rewarming [33, 38]. However, this study concluded that TH for severe TBI did not improve the neurologic outcome or risk of mortality [41].

The reason for the negative results obtained with TH for TBI may have been the heterogeneity of the study population, particularly pertaining to the age and degree of primary brain damage. Yamamoto et al. [42] reported an age limit of 50 years for TH to be effective in TBI. The subanalysis of BHYPO, according to computed tomography (CT) classification of the Traumatic Coma Data Bank on admission [43], showed that favorable outcomes at 6 months after injury in young patients (≤50 years of age) with evacuated mass lesions significantly increased from 33.3 % under fever control to 77.8 % under TH. Clifton [44] reported a similar post hoc analysis of two randomized clinical trials [38, 39] and showed that 41 % of patients requiring craniotomy for hematoma treated with early hypothermia experienced a poor outcome at 6 months after injury compared with 62 % of patients treated with late hypothermia or normothermia, a 34 % reduction. Induction of hypothermia to 35 °C before or immediately after craniotomy, with maintenance at 33 °C for 48 h thereafter, may improve outcomes of patients with severe TBI with surgically treated hematomas [44].

#### Fever control (normothermia)

Conversely, the subanalysis of BHYPO, according to CT classification of the Traumatic Coma Data Bank on admission [43], showed that patients with diffuse injury III who were treated with TH had significantly high mortality than those treated with fever control. Heterogeneity of the TBI population is also expressed using Abbreviated Injury Scale (AIS)-head scores, which describe TBI severity based on a combination of symptoms, mechanisms (blunt or penetrating), and radiographic findings (CT findings) [45]. The subanalysis of BHYPO, according to AIS-head scores on admission [46], showed that the fever control group demonstrated a significant reduction in mortality compared with the mild TH group (9.7 vs. 34.0 %, *p* = 0.02) pertaining to AIS 3 (serious) to 4 (severe) patients but not AIS 5 (critical) patients. Recently, the Eurotherm3235 trial study reported that TH plus standard care for elevated ICP provided a poor outcome for patients with TBI compared with the outcome provided by standard care alone [47].

TH influences all the organ systems, and any potential benefit should be balanced against possible side effects [48]. Several studies demonstrated that the rate of complications significantly increased during prolonged mild TH in patients with severe TBI [38–40, 49]. The management approach for patients between post-cardiac arrest and severe TBI may be different at temperatures below 35 °C because of multiple traumas associated with increased mortality [50]. This may contribute to coagulopathy, which usually occurs and persists for the first 24–48 h [51]. Therefore, fever control may be better than mild TH in terms of coagulopathy, which is specific for trauma patients. After the initiation of hypothermia in the BHYPO study subanalysis [46], platelet counts decreased more in the mild TH group than in the fever control group. In TH for TBI, coagulopathy contributed to a further degeneration of intracerebral lesion, which was the major cause of death.

#### Intracranial pressure and the timing of therapeutic hypothermia

The Eurotherm3235 trial reported that TH plus standard care for elevated ICP provided a poor outcome in patients with TBI compared to the outcome provided by standard care alone [47]. The key issue of TH for TBI is the timing of TH. The Eurotherm3235 results [47] may not be surprising because the elevation of ICP occurs because of various destructive processes following impact to the brain [52]. TH induction for subjects with already elevated ICP may be too late for suppressing these detrimental processes. In this study, significant ICP reduction could not be achieved in the TH group. Earlier induction of TH (before the elevation in ICP) may mitigate harmful physiological consequences and improve patient outcome. Another key issue of TH for TBI is the target. Early induction of TH, particularly for patients with evacuated intracranial hematomas, is reported to be beneficial according to a post hoc analysis of clinical trials in the USA [44] and Japan [43]. In addition, physiological effects are considerably different between 32 and 35 °C, and maintaining the temperature at 32 °C may cause more adverse events. Therefore, a detailed analysis on the introduction time and target temperature is desirable.

### Nonconvulsive status epilepticus and electroencephalography monitoring

#### Overview

Status epilepticus (SE) is defined as 5 min or more of (i) continuous clinical and/or electrographic or (ii) recurrent seizure activity without recovery (returning to baseline) between seizures [53]. The representative phenotype of nonconvulsive SE (NCSE), which is often observed in the intensive care setting, is acutely ill patients with severely impaired/altered mental status, with or without subtle motor movements (e.g., rhythmic muscle twitches or tonic eye deviation that often occurs during acute brain injury) [54–58]. NCSE in the ICU frequently follows uncontrolled or partially treated generalized convulsive SE (GCSE).

Frequency of NCSE diagnosis increases significantly after the implementation of continuous video-EEG monitoring in ICU [59]. NCSE is a relatively common condition among patients with unexplained altered mental status, with a prevalence of 8–37 % [54, 60]. In neuro ICU, nonconvulsive seizures have been reported in 18–34 % of patients who undergo EEG monitoring and 10 % of them are NCSE patients [61–63]. The incidence of NCSE in comatose survivors ranges from 12 to 24 % [48, 64, 65]. Seizures following out-of-hospital cardiac arrest have been linked to increased mortality [48, 64, 65]. NCSE should be diagnosed and treated rapidly to avoid significant morbidity and mortality [66]. A retrospective study of 100 NCSE patients identified the mortality rate to be 18 % [66].

#### Continuous electroencephalography monitoring

A consensus panel at the 4th London-Innsbruck Colloquium on SE and Acute Seizures held in Salzburg (2013) proposed working criteria for the EEG diagnosis of NCSE [67]. The American Clinical Neurophysiology Society (ACNS) published proposals for Standardized Critical Care EEG Terminology [68], which is now widely used and has high interrater agreement [69]. Salzburg Consensus Criteria for diagnosis of Non-convulsive Status Epilepticus (SCNC) implemented the ACNS definitions for rhythmic delta activity to avoid numerous false positives [70]. The ACNS criterion for fluctuation further marginally reduces false positives and in turn leads to a small loss of sensitivity.

There are many kinds of EEG which can be applied in the ICU. Conventional EEG, which is a gold standard for evaluating the neurologic function, only provides data over a period of 30 min and requires highly trained neurophysiologist to analyze it, whereas the aforementioned approach allows continuous monitoring by intensivists immediately after the patient is assigned a hospital bed [71].

Continuous EEG (cEEG) monitoring (over 24 h) is necessary to diagnose NCSE and to manage refractory SE [72]. cEEG monitoring should be initiated within 1 h of SE onset if ongoing seizures are suspected in all patients [53]. cEEG monitoring should be done for at least 48 h following acute brain insult in comatose patients to evaluate nonconvulsive seizures and 24 h after the cessation of electrographic seizures or during antiepileptic drug (AED) weaning trials [53].

Bedside simplified EEG monitoring, which comprised from two-channel system, is a kind of cEEG. The application of a two-channel simplified EEG has become widespread in clinical practice [73] with seizure detection sensitivity directly being correlated with the number of leads used. Using seven [74] and four leads [75] yielded sensitivities of 93 and 68 %, respectively. Single-channel EEG yields a sensitivity of 40 % [76]. The application of bedside simplified EEG for the diagnosis of NCSE may be limited to certain patients, particularly to those at a risk of consciousness disturbances following generalized convulsive status, recurrent coma, facial myoclonus, rapid involuntary eye movements, or aphasia [75].

Quantitative EEG may assist efficiently screening large amounts cEEG data [77, 78]. Amplified-integrated EEG (aEEG) monitoring is a well-developed type of quantitative EEG. aEEG system is widely used to predict the outcome of hypoxic ischemic encephalopathy in adults [79]. A bedside monitoring system combining aEEG and simplified EEG was designed for the early diagnosis of NCSE, which can then be confirmed by conventional EEG because of the inherent low sensitivity of the simplified EEG system [80]. Interpretation of aEEG and simplified EEG traces requires some training to recognize the patterns indicative of NSCE; these patterns are divided into “rhythmicity,” “spike and wave,” and “periodicity” [68].

### Subarachnoid hemorrhage

#### Advanced monitoring for circulation and delayed cerebral ischemia

The occurrence of delayed cerebral ischemia (DCI) after subarachnoid hemorrhage (SAH) increases poor neurologic outcome [81] and the risk of DCI during hypovolemia that is frequently observed in SAH [82]. Neurogenic pulmonary edema (PE), i.e., excess fluid accumulation in the lungs, is another complication observed in SAH [83]. Neurogenic PE can produce severe hypoxemia, thereby contributing to cerebral hypoxia in a brain that is already vulnerable to secondary injury [84].

In a recent randomized trial [85], physician-directed prophylactic triple-H administration was not associated with improved clinical outcomes or quantitative hemodynamic indicators for intravascular volume. Furthermore, the global end-diastolic volume index (GEDI), measured using transpulmonary thermodilution method-directed intervention studies, is warranted to better define management algorithms for SAH patients with the aim of preventing DCI. In a multicenter prospective cohort study, for GEDI on days 1–7 after SAH, the optimal range for fluid management according to the Cox proportional hazards model was suggested to be 822–921 ml/m^2^ for preventing the subsequent development of DCI and pulmonary edema [86], even though the event of myocardial stunning is not described. Mutoh et al. reported that in comparison with standard less-invasive hemodynamic therapy, early goal-directed therapy is beneficial for optimizing the complex SAH-induced hemodynamic changes during the therapy for DCI and in improving the prognosis of patients with poor World Federation of Neurosurgical Societies grade or coexisting cardiopulmonary complications [87].

Euvolemic fluid management is recommended in neurocritical care, including SAH, even though fluid balance [88] or central venous pressure [89] monitoring is not standardized for targeting. Fluid management may ameliorate neurologic outcomes and decrease mortality associated with SAH, particularly by avoiding hypovolemia and pulmonary edema.

#### Effect of neurocritical care/neuro-intensivists on outcome

Neurointensivists are physicians specially trained in neurocritical care. Neurocritical care is one of the fellowship subspecialties and board certified in the United Council for Neurologic Subspecialties in the USA. Neurointensivists assume the primary care role for his/her patients in the ICU, coordinating both the neurological and medical management of the patient.

The impact of neurocritical care/neuro-intensivists’ care on outcomes in patients with life-threatening neurological and neurosurgical illnesses has been reported by various authors [90–95]. In particular, the proven efficacy of a neurointensivist-managed neurocritical/intensive care unit for patients with aneurysmal SAH has been frequently reported. Neurointensivist-managed ICUs have reported positive efficacy and outcomes for SAH patients pertaining to their length of ICU stay [94, 96] and hospital discharge status (home, rehabilitation facility, nursing home, and death) [97]. However, there are no reported studies demonstrating efficacy via direct functional outcomes (i.e., good neurological outcomes at hospital discharge) [96–98].

For SAH, in severe cases [Hunt and Hess grade III–V subgroup], brain damage is severe and the efficacy of neurocritical care is not precisely evaluated. Samuels et al. reported that H&H grade I–III patients in a post-neurointensive care group were significantly more likely to be discharged home [97]. In addition, Knopf et al. reported that the availability of a neurointensivist (but not a neurocritical care unit) improved outcomes particularly in SAH patients with longer ICU and hospital stays [98]. Literature demonstrated that the stated efficacies of neurocritical care in SAH patients were limited to studies in the USA [1, 92, 94, 96–99].

Thus, neurointensivist management, particularly in SAH patients, outside the USA should be evaluated to demonstrate efficacies on brain damage-dependent neurological outcomes.

### Sepsis-associated brain dysfunction

#### Overview

Sepsis-associated encephalopathy (SAE), which has also been recently termed as sepsis-associated brain dysfunction (SABD), is a global brain dysfunction secondary to the systemic inflammatory response to cerebral perfusion and neuronal activity. It occurs because of infection in the body and in the absence of a direct infection of the central nervous system. Acute brain dysfunction commonly occurs during sepsis and typically develops early, often before the other organs are affected.

SAE involves a number of mechanisms, including neuroinflammation, wherein the interaction between cytokines and neurotransmitters, particularly acetylcholine, results in neuronal loss and alterations in cholinergic signaling. There is no clear evidence to explain in detail how inflammation reaches the brain during sepsis, but inflammation occurs in the CNS early and later after sepsis (both in laboratory animals and in humans). Moreover, the interaction occurs in the periphery, accelerating a type of immunosuppressive state.

Clinical, electrophysiological, and biochemical criteria are used to diagnose SAE. However, the inability to properly recognize the signs of SAE for diagnoses is a major ongoing problem because septic patients are usually sedated, which masks neurologic disturbances.

Although its diagnosis is not specific in biochemistry and imaging tests, it could potentiate severe outcomes, including increased mortality, cognitive decline, progressive immunosuppression, cholinergic anti-inflammatory deficiency, and metabolic and hydroelectrolyte imbalance. Therefore, bilateral communication between SAE and multiple peripheral organs (particularly the immune system) should be emphasized in sepsis management.

#### Examination to detect sepsis-associated brain dysfunction

Clinical symptoms of SAE are characterized by altered mental status, i.e., alteration in consciousness ranging from delirium or disorientation to coma, seizure, or focal neurological signs. However, SAE or SABD overlaps with delirium syndrome that is commonly associated with critical illness.

A recent systemic review showed that the incidence of EEG abnormalities during sepsis ranged from 12 to 100 % for background abnormalities and 6 to 12 % for triphasic waves [100]. The aforementioned EEG abnormalities, including epileptiform discharges or electrographic seizures, are associated with the presence and severity of SAE/SABD. There is some evidence supporting EEG use in the detection and prognostication of SAE [100]. A recent study of ICU patients (35 % with sepsis) found that time taken for burst suppression during coma was an independent predictor of the prevalence of and time to resolve post-coma/post-deep sedation ICU delirium [101]. Considering the possible link between excess sedation use and SABD, the avoidance of burst suppression using EEG monitoring may attenuate brain dysfunction in sepsis patients.

Magnetic resonance imaging (MRI) in the acute phase (7–14 days), including leukoencephalopathy and ischemic stroke, is associated with a poor prognosis in SAE [102]. Other reports showed that these findings were hardly specific to SAE [103], and a recent SAE case with a favorable neurological outcome was reported in which the authors showed severe encephalopathy with extensive white matter lesions [104]. Taken together, it is controversial whether MRI imaging changes noted in the acute phase (7–14 days) are helpful in detecting SAE. A recent study showed that a longer duration of delirium was strongly associated with a greater extent of brain atrophy as well as white matter disruption on advanced MRI scan performed at hospital discharge and 3-month follow-up [105].

Using transcranial Doppler ultrasound to estimate cerebral blood flow, a recent report showed that alterations in the autoregulation of cerebral blood flow can persist for several days in >40 % patients with septic shock [106] and that cerebral vasoconstriction on the first day of sepsis diagnosis implied the development of neurological symptoms independent of the age and APACHE II score [107].

As serum biomarker for prognosis, serum S100beta concentrations were significantly higher in patients with sepsis-associated brain dysfunction than in normal controls and correlated directly to GCS [108].

The diagnosis of SAE or SABD usually depends on case history and clinical symptom and may be affected by sedation and intubation. For early diagnosis and evaluation of severity of SAE or SABD, neuromonitoring using EEG, MRI, transcranial Doppler ultrasound, and serum biomarker may be helpful.

## Conclusions

Neurocritical care provides comprehensive neurological support for patients with life-threatening neurological and neurosurgical illnesses by integrating and balancing the management of both the brain and the other organs. New therapies that address the underlying pathophysiology are required to improve neurologic outcomes.
