# Erratum to: Neurocritical care update

**DOI:** 10.1186/s40560-016-0168-x

**Published:** 2016-07-25

**Authors:** Yasuhiro Kuroda

**Affiliations:** Department of Emergency, Disaster, and Critical Care Medicine, Faculty of Medicine, Kagawa University, 1750-1, Ikenobe, Miki, Kita, Kagawa 761-0793 Japan

## Erratum

Unfortunately, the original version of this article [1] contained several errors:

- Reference 24 in the original article (reference [2] in this erratum) should have been “Brophy GM, Human T, Shutter L. Emergency neurological life support: pharmacotherapy. Neurocrit Care. 2015;23(Suppl 2):S48–68” instead of “Badjatia N, Kowalski RG, Schmidt JM, Voorhees ME, Claassen J, Ostapkovich ND, Presciutti M, Connolly ES, Palestrant D, Parra A, et al. Predictors and clinical implications of shivering during therapeutic normothermia. Neurocrit Care. 2007;6(3):186–91”.
- Reference 25 in the original article (reference [3] in this erratum) should have been “Badjatia N, Strongilis E, Gordon E, Prescutti M,Fernandez L, Fernandez A, Buitrago M, Schmidt JM, Ostapkovich ND, Mayer SA. Metabolic impact of shivering during therapeutic temperature modulation: the Bedside Shivering Assessment Scale. Stroke. 2008 Dec;39(12):3242-7. doi: 10.1161/STROKEAHA.108.523654. Epub 2008 Oct 16” instead “Brophy GM, Human T, Shutter L. Emergency neurological life support: pharmacotherapy. Neurocrit Care. 2015;23(Suppl 2):S48–68”.
- Accordingly, the caption of Figure 1 that cited reference 24 and 25 was wrong too, and it should have been “Anti-shivering protocol example and the bedside shivering assessment scale. IV intravenous, PT per feeding tube, ECG electrocardiogram. Modified from Brophy [24] and Badjatia [25] with permission” instead of “Anti-shivering protocol example and the bedside shivering assessment scale. IV intravenous, PT per feeding tube, ECG electrocardiogram. Modified from Brophy [25] and Badjatia [24] with permission”.

The references have been corrected in the original article and are also correctly included in this erratum.
